# Study on *Oleum cinnamomi* Inhibiting *Cutibacterium acnes* and Its Covalent Inhibition Mechanism

**DOI:** 10.3390/molecules29133165

**Published:** 2024-07-03

**Authors:** Huayong Peng, Chenliang Chu, Lu Jin, Jianing Zhang, Zilei Yang, Longping Zhu, Depo Yang, Zhimin Zhao

**Affiliations:** 1 School of Pharmaceutical Sciences, Sun Yat-sen University, Guangzhou 511400, China; penghy29@163.com (H.P.); jinlu5@mail.sysu.edu.cn (L.J.); zhangjn65@mail2.sysu.edu.cn (J.Z.); 273169828@163.com (Z.Y.); zhlongp@mail.sysu.edu.cn (L.Z.); 2 School of Pharmaceutical Sciences, Jishou University, Jishou 416000, China; 3 School of Food and Pharmaceutical Engineering, Zhaoqing University, Zhaoqing 526060, China; ccl15918621025@163.com

**Keywords:** *Cutibacterium acnes*, Oleum cinnamomi, antibacterial, metabolomics, covalently modifies, ABC transporter ATP-binding protein, NADH-quinone oxidoreductase

## Abstract

*Oleum cinnamomi* (OCM) is a volatile component of the *Cinnamomum cassia* Presl in the Lauraceae family, which displays broad-spectrum antibacterial properties. It has been found that OCM has a significant inhibitory effect against *Cutibacterium acnes* (*C. acnes*), but the precise target and molecular mechanism are still not fully understood. In this study, the antibacterial activity of OCM against *C. acnes* and its potential effect on cell membranes were elucidated. Metabolomics methods were used to reveal metabolic pathways, and proteomics was used to explore the targets of OCM inhibiting *C. acnes*. The yield of the OCM was 3.3% (*w*/*w*). A total of 19 compounds were identified, representing 96.213% of the total OCM composition, with the major constituents being phenylpropanoids (36.84%), sesquiterpenoids (26.32%), and monoterpenoids (15.79%). The main component identified was trans-cinnamaldehyde (85.308%). The minimum inhibitory concentration (MIC) and minimum bactericidal concentration (MBC) of OCM on *C. acnes* were 60 µg/mL and 180 µg/mL, respectively. The modified proteomics results indicate that cinnamaldehyde was the main bioactive ingredient within OCM, which covalently modifies the ABC transporter adenosine triphosphate (ATP)-binding protein and nicotinamide adenine dinucleotide (NADH)-quinone oxidoreductase, hindering the amino acid transport process, and disrupting the balance between NADH and nicotinamide adenine dinucleoside phosphorus (NAD^+^), thereby hindering energy metabolism. We have reported for the first time that OCM exerts an antibacterial effect by covalent binding of cinnamaldehyde to target proteins, providing potential and interesting targets to explore new control strategies for gram-positive anaerobic bacteria.

## 1. Introduction

*Cinnamomum cassia* Presl is a plant of the *Cinnamomum* genus of the Lauraceae family that is indigenous to China and extensively grown in tropical and subtropical areas. *Oleum cinnamomi* (OCM) was isolated from the dried branches and leaves of *Cinnamomum cassia* Presl by steam distillation and was commonly used in traditional Chinese medicine. Modern studies have confirmed that *Cinnamomum cassia* has a wide range of pharmacological effects, including antitumor, anti-inflammatory, analgesic, anti-diabetic, anti-obesity, antibacterial, and antiviral, cardiovascular protective, cytoprotective, neuroprotective, immunoregulatory effects, anti-tyrosinase activity, and other effects [1]. OCM is documented as an antibacterial drug in traditional Chinese medicine works such as Supplement to Compendium of Materia, Annals of Traditional Chinese Medicine in Guangxi, and Chinese Pharmacopoeia 2020. The Chinese pharmacopoeia uses gas chromatography to standardize the quality of OCM, ensuring that the cinnamaldehyde content is not less than 75.0%. Recent research indicates that OCM has antibacterial activity against various bacteria such as *Escherichia coli*, *Staphylococcus aureus*, *Salmonella bongori*, *Bacillus cereus*, *Enterococcus faecalis*, *Streptococcus pyogenes*, *Pseudomonas aeruginosa*, etc. [2,3,4,5]. It is also one of the spices that can inhibit the growth of fungi such as *Candida* and *Aspergillus niger*. Indians often use it as an anti-mildew agent during food storage. Therefore, OCM has excellent potential for development in the food industry and medical field.

*Acne* is a persistent inflammatory skin disease that affects the hair follicle sebaceous glands and is prone to occur in adolescents. It can cause damage to the patient’s skin, resulting in pigmentation and scars, seriously affecting the patient’s appearance, physical health, and mental well-being. *Cutibacterium acnes* (*C. acnes*) has been identified as the primary cause of acne. Macrolides and tetracyclines are considered one of the most effective acne treatments as they can alleviate the pathogenesis of acne through their antibacterial effects against the proliferation of *C. acnes* within the sebaceous follicle.

However, long-term oral administration of macrolides or tetracyclines in the treatment of acne not only increases antibiotic-resistant *C. acnes* but also leads to an increase in antibiotic-resistant *Staphylococcus* in the patient. This highlights the challenges associated with the long-term treatment of *C. acnes* and the prevention of relapse [6]. The detection rate of drug-resistant *C. acnes* strains, which are resistant to antibiotics such as tetracycline, macrolides, and β-lactam antibiotics, has been steadily increasing due to the widespread use and, in some cases, abuse of antibiotics [7,8]. Consequently, new targets for combating antibiotic-resistant *C. acnes* and developing safe and nonresistant anti-*C. acnes* treatments are currently under exploration. Acne medications have significant implications for *acne* treatment and the development of acne-removing cosmetics [9,10].

OCM has potent inhibitory activity against *C. acnes* [2,3,4] and can be used as an antibacterial ingredient in acne-removing cosmetics. Previous studies have shown that cinnamaldehyde in *Cinnamomum cassia* Presl can disrupt the lipid metabolism of *Escherichia coli* and its cell membrane integrity. Methoxycinnamaldehyde can deplete glutathione in *Staphylococcus epidermidis* and disrupt redox balance and amino acid metabolism. Bioinformatics analysis reveals that OCM primarily inhibits biological activity through aldehyde toxicity [11], acid stress [12], oxidative stress [13], disrupting protein translation [14], carbohydrate metabolism [15], and energy metabolism [16]. However, the target and molecular mechanism of OCM inhibiting *C. acnes* is still unclear, which hinders its potential application in acne-removing cosmetics.

In recent years, the rapid development of chromatography combined with high-resolution mass spectrometry has provided strong support for studying drug mechanisms and targets. This study utilized gas chromatography–mass spectrometry (GC–MS) and liquid chromatography–mass spectrometry (LC–MS) to investigate the antibacterial mechanism of OCM against *C. acnes* after short-term drug exposure. The metabolomics findings were integrated with other biochemical approaches to gain a more comprehensive and systematic understanding of the potential antibacterial targets of *C. acnes*.

## 2. Results

### 2.1. Chemical Compositions of the OCM

The yield of the OCM was 3.3% (*w*/*w*). The chemical compositions of the OCM are listed in Table 1 by GC–MS analysis. Nineteen compounds accounted for 96.213% of the total OCM components. Chemical components of OCM, comprising more than 1% of the total content, were considered the main components. Trans-cinnamaldehyde was the most abundant constituent, with a relative percentage of 85.308%, consistent with the previous literature [17]. The next top three were 2-methoxycinnamaldehyde (2.145%), benzene propanal (2.122%), and benzaldehyde (1.276%). The identified components of the OCM can be categorized into seven groups: phenylpropanoids, sesquiterpenoids, monoterpenoids, aliphatic compounds, aromatic alcohols, aromatic aldehydes, and bicyclic sesquiterpenes. The proportions of the first three groups are 36.84%, 26.32%, and 15.79%, respectively. The combined content of the remaining four groups is 21.04%.

### 2.2. Antibacterial Activity of OCM against C. acnes

**Figure 1:**
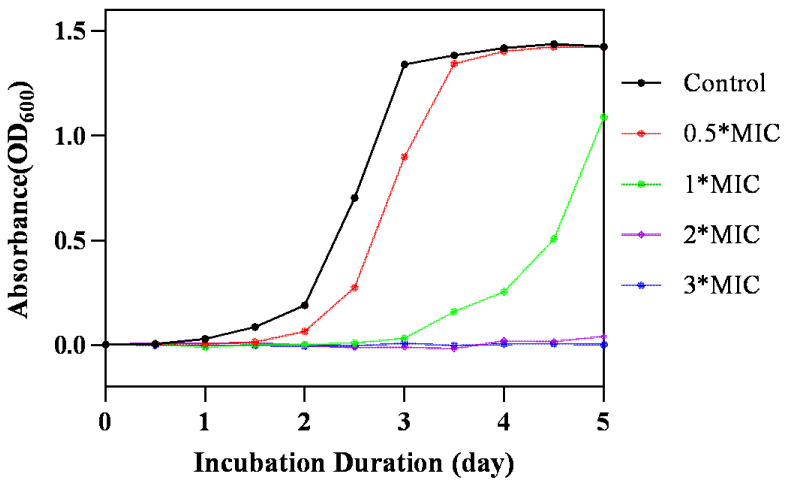
Effect of OCM on the growth curve of *C. acnes* within 5 days. The bacteria were treated with solvent (control) and different concentrations of OCM (0.5*MIC, 1*MIC, 2*MIC, and 3*MIC).

The minimum inhibitory concentration (MIC) and minimal bactericidal concentration (MBC) were measured, and the bacterial growth curves of bacteria exposed to various concentrations of OCM were recorded. MIC and MBC values of OCM against *C. acnes* were 60 µg/mL and 180 µg/mL, respectively, with tetracycline used as a positive control. The MIC of tetracycline was 1.85 µg/mL, and the MBC was 5.55 µg/mL. The optical density (OD_600_) started to increase after 1 day of incubation in the control group, indicating that the solvent control did not affect the proliferation of *C. acnes*. As shown in Figure 1, *C. acnes* reached the logarithmic growth stage within 2 days and entered the plateau stage within 3 days. When the concentration of OCM is 0.5*MIC, the exponential growth period of *C. acnes* is postponed, and when the concentration of OCM is 2*MIC or 3*MIC, *C. acnes* stops growing, and a bactericidal effect is achieved. This experimental result is consistent with the literature report [18]. The antibacterial activities of trans-cinnamaldehyde, 2-methoxy cinnamaldehyde, benzene propanal, and benzaldehyde, the main constituents within OCM, were tested, and the results from our current study revealed that the MICs of trans-cinnamaldehyde and 2-methoxy cinnamaldehyde were 50 μg/mL each. In contrast, the MICs of benzene propanal and benzaldehyde are more than 200 μg/mL or ineffective. As a result, cinnamaldehyde had an equivalent antibacterial activity to that of the OCM, indicating that trans-cinnamaldehyde and 2-methoxy cinnamaldehyde are probably the main antibacterial agents.

**Figure 2:**
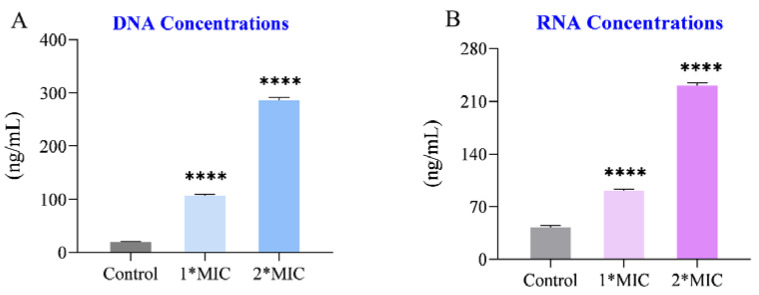
OCM induced the leakage of (**A**) DNA and (**B**) RNA from *C. acnes. C. acnes* in the exponential growth phase was treated with OCM at 1*MIC and 2*MIC for 6 h, while the 0.1% solvent (n-hexane/ isopropanol = 1:1) was used as a control. The leakages of DNA and RNA were quantified by measuring the absorbance at 260 and 230 nm using a NanoDrop spectrophotometer. The pooled data are presented as mean ± SEM of at least three independent experiments. **** *p* < 0.0001.

In addition, the permeability of the cell membrane is measured by the leakage of deoxyribonucleic acid (DNA) and ribonucleic acid (RNA) into the supernatant. As depicted in Figure 2, the DNA and RNA content of *C. acnes* subjected to OCM treatment showed a significant increase compared to those of the control group. This indicates that the permeability of *C. acnes* increased after OCM treatment.

### 2.3. Scanning Electron Microscope (SEM) and Transmission Electron Microscope (TEM)

**Figure 3:**
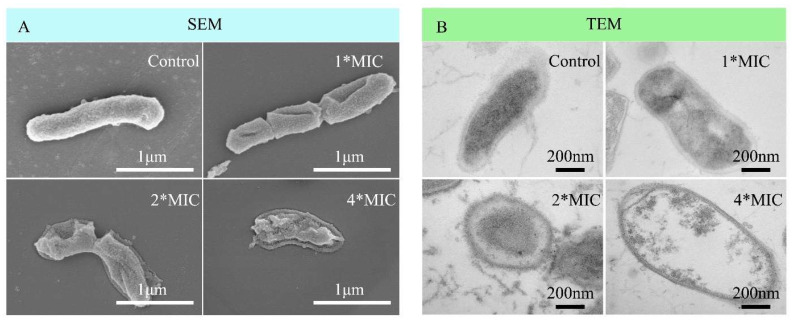
OCM altered the morphology and ultrastructure of *C. acnes*. After treatment with solvent or different concentrations of OCM (1*MIC, 2*MIC, and 4*MIC) for 6 h, the bacteria were collected, fixed, and prepared for electron microscopy analysis. The images from SEM and TEM are shown in panels (**A**,**B**), respectively.

The morphological changes of *C. acnes* before and after OCM treatment were analyzed using SEM and TEM. Figure 3 shows that untreated cells exhibited intact cell structure, including thick cell walls and uniform cytoplasm. After OCM treatment, cell wall damage, changes in cytoplasmic density, and cell swelling were observed. The observed changes in bacterial structure indicate that antibacterial components in OCM can enter cells, affecting cell walls, protoplasts, and intracellular proteins.

### 2.4. Metabonomics

#### 2.4.1. Effect of OCM on Metabolic Profiles of *C. acnes*

**Figure 4:**
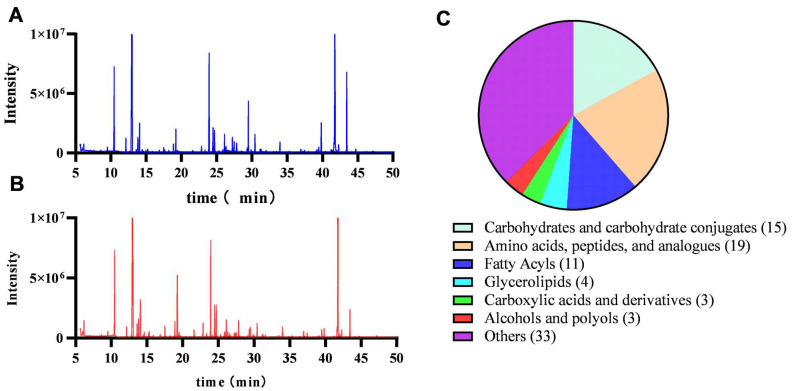
Metabolic profiling of *C. acnes* before and after EO treatment. (**A**) Typical total ion chromatograms of the metabolic profiles of *C. acnes* with solvent (control) derived from GC–MS. (**B**) Typical total ion chromatograms of the metabolic profiles of *C. acnes* with OCM (1*MIC). (**C**) Proportions and types of differential metabolites.

GC–MS was used to study the impact of OCM on bacterial metabolism. A total of 88 metabolites were identified. According to the results of Figure 4A,B, there was a significant difference in metabolite abundance between the MIC and control groups. As shown in Figure 4C, all the metabolites can be divided into seven categories, including carbohydrates and carbohydrate conjugates (17.0%), amino acids (21.6%), fatty acyls (12.5%), glycerolipids (4.6%), carboxylic acids (3.4%), alcohols and polyols (3.4%), and others (37.5%).

#### 2.4.2. Differential Metabolites Analysis

**Figure 5:**
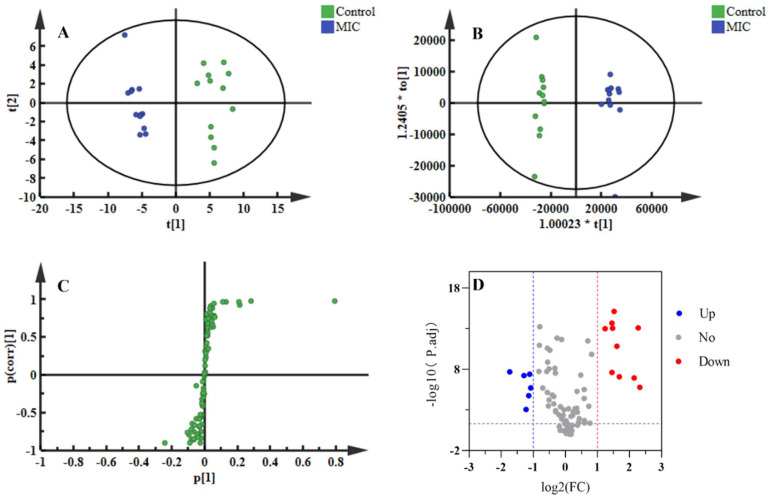
Metabonomics statistical analysis. (**A**) PCA score plot (R2X = 0.752, Q2 = 0.434) (**B**) OPLS-DA score plot (R2X = 0.431, R2Y = 0.967, Q2 = 0.953). (**C**) S-plots from the OPLS-DA model; the abscissa indicates the covariance and the ordinate indicates the correlation. (**D**) Volcano plot; red dots represent the increase in metabolite abundance, and blue dots represent the decrease in metabolite abundance (vs. control).

Multivariate statistical analysis was used to investigate the metabolic differences between the OCM treatment group (1*MIC) and the control group. Principal component analysis (PCA) analyzed metabolites, and there was a clear separation between the control and treatment groups on the score plot (Figure 5A). A supervised orthogonal partial least squares discriminant analysis (OPLS-DA) model was established to compare metabolic changes between the 1*MIC group and the control group. The Q2 value of this model is 0.953, indicating that it has high validation predictability and can be used further to screen differential metabolites (Figure 5B). In the S-plot of OPLS-DA (Figure 5C), each spot represents a compound, and the farther away from the origin, the more significant its contribution to the clustering of the two groups. The contribution is expressed by the variable importance of the project’s value (VIP). Eighty-eight metabolites were selected using a student’s *t*-test to determine the significant difference between the two groups (*p*-value adjusted (p.adj) < 0.05), and fold change (FC) was calculated to measure the extent of up/down-regulation of metabolites. Only when the p.adj was lower than 0.05 and the FC was lower than 0.5 or higher than 2 could metabolites be considered differential metabolites, as shown in Figure 5D. Before and after OCM administration, a total of 17 endogenous metabolites were identified as differential metabolites and categorized. Among these, 6 metabolites decreased while 11 increased, and they have been designated as biomarkers.

#### 2.4.3. Enrichment of Metabolic Pathway

**Figure 6:**
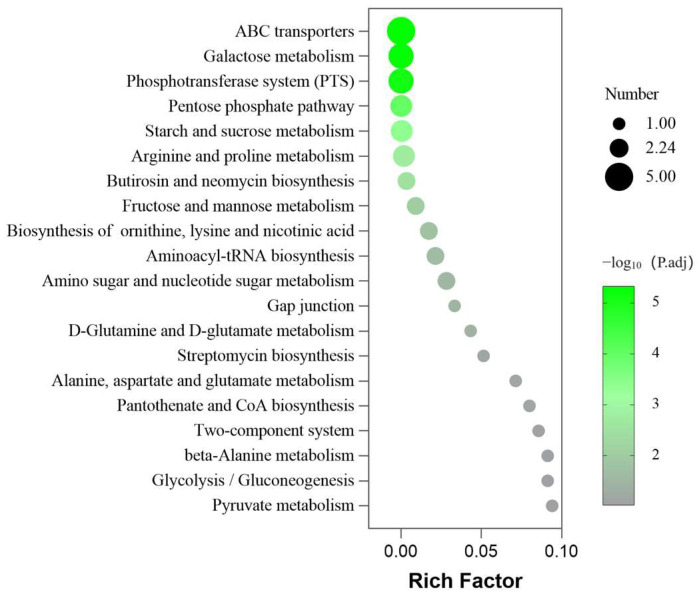
Kyoto Encyclopedia of Genes and Genomes (KEGG) enrichment analysis bubble chart of metabolic pathways. The x-axis represents the rich factor (the number of differential metabolites enriched in the pathway). The y-axis represents the name of KEGG pathways. The size of the bubble represents the number of the differential metabolites, and the color represents *p*-values.

Pathway enrichment analysis was conducted on 17 differential metabolites, resulting in 20 metabolic pathways (p.adj < 0.05), as shown in Figure 6. The top six pathways were ABC transporters, galactose metabolism, phosphotransferase system, pentose phosphate pathway, starch and sucrose metabolism, and arginine and proline metabolism. It was found that L-glutamic acid, D-glucose, D-fructose, L-proline, D-mannose, gluconic lactone, lactose, isomaltose, D-gluconic acid, and hydroxyproline were the critical intermediates in the metabolic pathway.

### 2.5. Identification of Modified Proteins

Cinnamaldehyde, the main component of OCM, can covalently bind to nucleophilic residues of proteins, including cysteine, histidine, and lysine, especially thiol residues, due to the high reactivity present in its structure α,β-unsaturated carbonyl pharmacophores [19,20,21,22]. We speculated that covalent modification of proteins may be one of the mechanisms of antibacterial activity. To identify which proteins cinnamaldehyde can bind to, a modified proteomics method was used to analyze cinnamaldehyde-modified proteins. The raw data were searched to identify peptides, in which two modification modes from Cin1 to Cin2 were added as variable modifications to cysteine, aspartate, histidine, and lysine residues.

**Figure 7:**
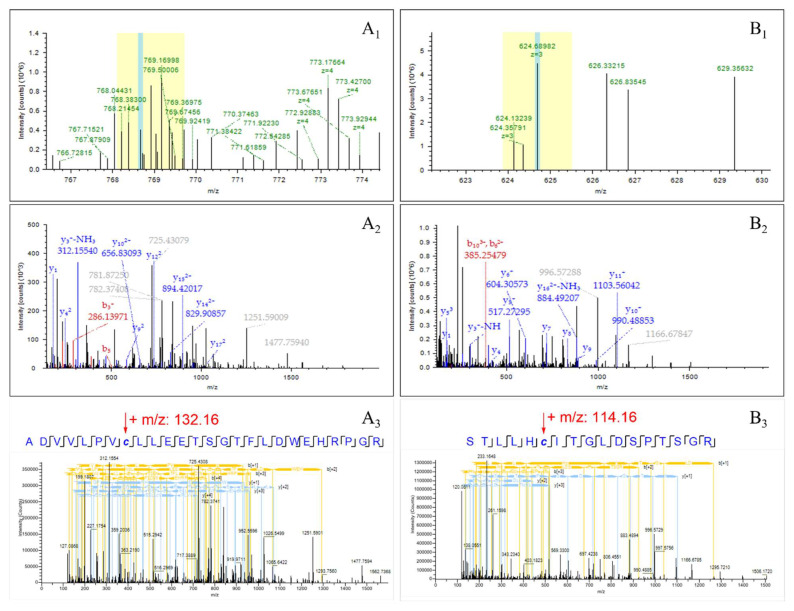
The MS and MS/MS spectra for the cinnamaldehyde-modified peptides. *C. acnes* was exposed to 1*MIC of OCM for 4 h, and the proteins of *C. acnes* were extracted for proteomic analysis. (**A1**,**A2**) represent the primary and secondary mass spectra of the cinnamaldehyde-modified peptide sequence ADVVLPVCLLEETSGTFLDWEHRPGR, which belongs to the protein A0A085B5J9. (**A3**) displays the peptide consensus view of b ions and y ions in the secondary mass spectrum. (**B1**,**B2**) display the primary and secondary mass spectra of the cinnamaldehyde-modified peptide segment, STLLHCITGLDSPTSGR, which belongs to the protein A0A828UH99 (**B3**), showing a peptide consensus view of b ions and y ions of the peptide in (**B2**).

Two modifications were successfully detected in OCM-treated bacteria and identified by characteristic MS/MS fragmentation ions. At the same time, no corresponding products were observed in the control group (Figure 7). Two modified proteins were identified from OCM-treated bacteria. Cysteine residues were found to be modified by the modification mode of Cin1 and Cin2. The modified peptide AdvLPVCLLEETSGTFLDWEHRPGR produced a molecular ion of b84+ at *m*/*z* 586.2968, which increased by 132.16 Da compared with the unmodified peptide (Figure 7A1–A3). Compared with the unmodified peptide (STLLHCITGLDSPTSGR), it was also observed that the fragmentation ion of b62+ increased by 114.16 Da, suggesting that cysteine should be modified by Cin2 (Figure 7B1–B3). In addition, the mass of other fragmentation ions of y2 and y5-y7 was the same as that of the unmodified peptide, indicating that the peptide belongs to STLLHCITGLDSPTSGR. These results confirm that cinnamaldehyde can covalently modify cysteine residues of proteins. The highly credible Cin1 and Cin2 modifications, accurate peptide amino acid sequences, and corresponding proteins were identified, providing strong evidence that cinnamaldehyde can covalently modify proteins in vivo.

These two peptides, found to be expressed in cinnamaldehyde modification compared with untreated cells, belong to two proteins: A0A085B5J9 and A0A828UH99, respectively. A0A085B5J9 functions as an NADH-quinone oxidoreductase, facilitating the transfer of electrons from NADH to quinones within the respiratory chain by utilizing FMN and iron–sulfur (Fe–S) centers. This redox reaction is coupled with proton translocation, preserving redox energy in the proton gradient. In contrast, A0A828UH99 is an adenosine triphosphate (ATP)-binding cassette (ABC) protein, which aligns well with the enriched metabolic pathway ABC transporter in metabolomics. The ABC protein is involved in ATP binding and ATP hydrolysis activity. These findings suggest that the covalent connection of critical proteins in the cell inhibits growth and disrupts vital cellular functions, subsequently leading to variations in internal cellular components in the cytoplasm and disruption of metabolic pathways, ultimately causing rapid bacterial cell death [23].

### 2.6. Changes in Amino Acids Content over Time

**Figure 8:**
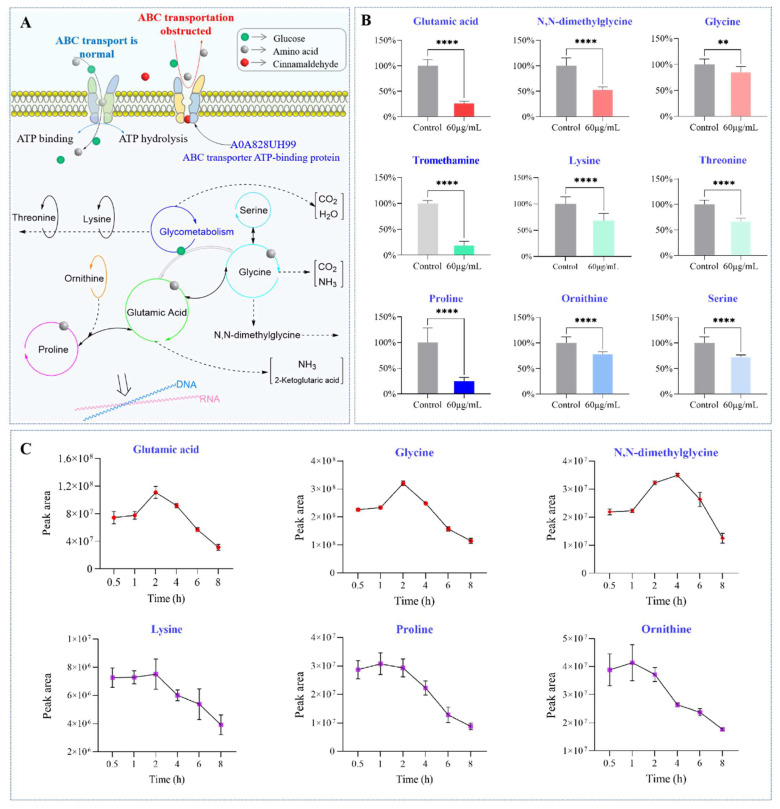
The effect of cinnamaldehyde covalently modifying ABC transporters on amino acids in bacteria. (**A**) Schematic diagram of ABC transporter proteins involved in transmembrane transport and metabolism of amino acid (**B**) Peak areas of amino acids of 1*MIC group and control group. *C. acnes* in the logarithmic growth phase was treated with OCM at 1*MIC for 6 h, and solvent was used as the vehicle control. The peak areas of indicated metabolites were quantified by GC–MS-based metabolomics. After normalizing the measured areas to the control group, the fold changes were used for quantification. (**C**) The contents of amino acid in bacterial cells after treatment with OCM. The peak areas of indicated amino acids were quantified by GC–MS-based metabolomics. The pooled data are presented as mean ± SEM of at least three independent experiments. ** *p* < 0.01, **** *p* < 0.0001.

ABC transporter is involved in transport processes with amino acids [24]. Figure 8A shows the schematic diagram of the ABC transporter transport mechanism. Utilizing the energy released by binding and hydrolyzing ATP drives its structure to alternate between different configurations, transporting substrates. The transport process is also obstructed when ATP binding and hydrolysis processes are inhibited. Amino acids are the fundamental building blocks of proteins and play an essential role in regulating material metabolism and information transmission in living organisms. Proline can be converted to glutamic acid and ornithine. First, double bonds are formed by proline oxidase, followed by ring opening with water to form glutamic acid g semialdehyde, which is oxidized to glutamic acid with NAD^+^. Glycine can first be metabolized to form serine, which is then converted to pyruvate and enters the carboxylic acid cycle. It can also undergo gluconeogenesis and be converted back into sugar. Glycine can also be converted into purine bases, creatine, and porphyrins. Glutamate can be metabolized through glycolysis for energy supply. It can also be converted into various amino acids, including ornithine, serine, glycine, etc. Threonine is a non-essential amino acid. L-threonine biosynthesis is directed towards aspartic acid metabolism through the glycolysis (EMP) and tricarboxylic acid (TCA) cycle pathways, as well as the hexose monophosphate pathway (HMP) pathway and the biosynthesis of lysine and valine. Multiple amino acids can be converted into each other or participate in bacteria’s material and energy metabolism. As shown in Figure 8B, we found that the content of all amino acids decreased, including tromethamine, glutamic acid, *N*,*N*-dimethylglycine, glycine, lysine, ornithine, proline, threonine, and serine, indicating the amino acid transport process was affected. Afterward, we studied the dynamic changes in amino acid content in bacteria after OCM treatment. The content of amino acids exhibited a trend of increase and then decrease (glutamic acid, *N*,*N*-dimethylglycine, glycine, proline) or a slight increase or no increase and then decrease (tromethamine, lysine, ornithine, threonine, and serine) with the time shown in Figure 8C. The maximum value appears approximately 4 h after administration. Amino acid dynamic analysis shows that the initial concentrations of glycine, proline, and glutamic acid increase during administration, confirming the existence of ABC transport. The non-increase in serine may be due to the amino acid not being transported but obtained through other pathways.

### 2.7. Target Verification: The NADH/NAD^+^ Ratio and Malate Dehydrogenase

**Figure 9:**
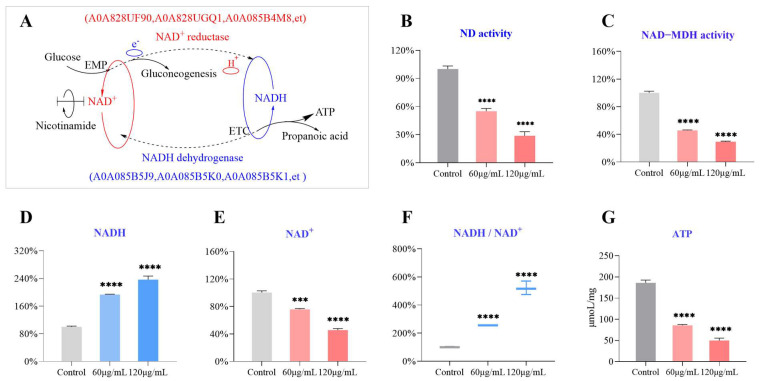
OCM affects the redox homeostasis and energy metabolism of *C. acnes*. (**A**) Schematic diagram of NADH quinone oxidoreductase participating in the conversion of NAD^+^ and NADH. The activities of NADH dehydrogenase and NAD-dependent MDH enzyme are shown in (**B**,**C**), respectively. The content of NADH, NAD^+^, NADH/NAD^+^ ratio, and ATP level in *C. acnes* cells are shown in panels (**D**–**G**), respectively. The pooled data are presented as mean ± SEM of at least three independent experiments. *** *p* < 0.001, **** *p* < 0.0001.

NAD (H) is a coenzyme that widely regulates biological processes such as cell growth, differentiation, energy metabolism, and apoptosis, as shown in Figure 9A. NAD^+^ is the primary hydrogen acceptor of EMP, and the generated NADH is transferred through the respiratory electron chain. While producing ATP, NADH is regenerated into NAD^+^ under the catalysis of dehydrogenase. The oxidation reactions of the three significant sugar, fat, and protein metabolites are mostly completed through this system. The levels of NAD (H) content and NADH/NAD^+^ ratio can be used to evaluate the strength of glycolysis. Under hypoxic conditions, elevated ratios of NADH/NAD^+^ result in the deactivation of multiple dehydrogenases, thereby inhibiting the glycolytic process. The colorimetric detection results of NAD^+^, NADH, and NADH/NAD^+^ ratios in bacterial cells treated with OCM are shown in Figure 9B. Compared with the control group, OCM decreased cellular NAD^+^ levels while significantly increasing NADH and the NADH/NAD^+^ ratio (*p* < 0.05). NADH dehydrogenase activity and the change in intracellular ATP content were determined. NADH dehydrogenase activity and ATP levels decreased significantly. The results are shown in Figure 9B–G. After treatment with OCM, the activities of NADH dehydrogenase in the 1*MIC and 2*MIC groups were reduced by approximately 46.2% and 71.0%. Additionally, the ATP levels in the 1*MIC and 2*MIC groups exhibited reductions of 52.8% and 73.6%, respectively. Furthermore, the activity of the NAD-dependent malate dehydrogenase (MDH) enzyme significantly decreases with the increase of OCM concentration, confirming the decrease in NAD content from another perspective. When the NADH/NAD^+^ ratio in the matrix is high, it will cause a decrease in respiratory chain electron transfer function and thus affect the cell’s ATP synthesis ability, leading to a decrease in ATP levels. In these cases, bacteria are in an oxidative stress state [25], ultimately leading to cell necrosis or apoptosis. A0A085B5J9 is a NADH-quinone oxidoreductase. The cinnamaldehyde in OCM inhibits NADH quinone oxidoreductase activity by covalently modifying A0A085B5J9 to block oxidative phosphorylation, limit ATP production, and thus prevent the growth of *C. acnes*.

## 3. Materials and Methods

### 3.1. Materials

*C. acnes* (ATCC 6919) strain was obtained from the Guangdong Microbial Strain Preservation Center. The mediums of brain heart infusion broth (BHI) media were purchased from HuanKai Microbial (Guangdong, China). Adonitol and *N*-methyl-*N*-trimethylsilyltriflfluoroacetanamide (MSTFA) were acquired from Sigma-Aldrich (St. Louis, MO, USA); AnaeroPack (Japan Mitsubishi MGC, Tokyo, Japan) was utilized to create hypoxic conditions. All other reagents were either imported or of analytical grade and were used in their original state without any purification.

### 3.2. Preparation of OCM from Cinnamomi ramulus

Plant Materials. The branches and leaves of *Cinnamomi ramulus* were collected in Deqing District, Zhaoqin City, Guangdong Province, in mid-May 2022. They were identified by Professor Depo Yang of Sun Yat-sen University, and the voucher specimen has been deposited in the herbarium of Sun Yat-sen University (specimen number: CAP220515). The plant materials were dried at room temperature in room protected from direct sunlight. The dried plant raw material was stored in bags.

We took the dried plant materials, cut them up, ground them, and then passed them through a 60-mesh sieve. A total of 100 g of *Cinnamomi ramulus* was placed in a 2000 mL round-bottom flask, and 1000 mL of distilled water and some zeolite were added. The mixture was subjected to steam distillation for 4 h at 100 °C using a Clevenger apparatus (Guangzhou, China). After distillation, the OCM was collected and dehydrated with anhydrous sodium sulfate for 24 h. The obtained OCM was measured three times, and its quantity was expressed per mass of dry plant material (g/g). The resulting pale-yellow OCM with a characteristic odor was stored at 4 °C in an amber vial until further analysis [17].

### 3.3. GC–MS Detection of OCM

We dissolved OCM in chromatographic-grade n-hexane at a ratio of 1:200 (*v*/*v*), shook thoroughly to dissolve using a Vortex oscillator (Ika VORTEX3, Guangzhou, China), filtered with a 0.22 μm microporous filter membrane, and conducted GC–MS (Thermo Scientific Trace DSQ II, Waltham, MA, USA) detection following the reference parameters [25]. A DB-5H column was used with helium as the carrier gas. Programmed to 220 °C at a rate of 5 °C/min and held for 15 min. Record mass spectrometry in 70 eV electron collision mode. Mass spectrometry data: 35–300 *m*/*z* full scan. Based on the National Institute of Standards and Technology (NIST) 2017 mass spectrometry library, the components of OCM are identified by comparing the obtained retention index (RI) with existing data in the literature.

### 3.4. The Antibacterial Activity of OCM against C. acnes

We detected the MIC and MBC values of OCM against *C. acnes* using the microbroth dilution method recommended by the Clinical and Laboratory Standards Institute 2017. In short, 50 µL of the bacterial suspension diluted to 1 × 10^5^ CFU/mL was inoculated in a 96-well plate and treated with different concentrations of OCM ranging from 15 to 1920 µg/mL, while 0.1% solvent (n-hexane/isopropanol = 1:1) was used as the vehicle control, and tetracycline was used as the positive control (concentrations ranging from 0.5 to 8 µg/mL). The plate was then incubated under anaerobic conditions at 37 °C for 24 h. After incubation, the MIC was determined as the lowest concentration (in µg/mL) of OCM that showed inhibitory effects on the growth of *C. acnes*. To determine the MBC, 100 µL of medium from each well was transferred and seeded on reinforced Clostridium agar (RCA) plates, and no visible bacterial growth was observed in the medium. After 72 h of culturing, the number of viable organisms was determined. A solvent control at the same concentration was included to eliminate its influence on bacterial growth.

### 3.5. Growth Assay

According to the literature [26], the logarithmic phase of *C. acnes* was resuspended with fresh medium to a concentration of 10^5^ CFU/mL. A specific amount of OCM was added to achieve concentrations of 0.5*MIC, 1*MIC, 2*MIC, and 3*MIC. The solvent was used as a vehicle control. Incubate at 150 rpm for five days at 37 °C while monitoring the OD_600_ value.

### 3.6. Determination of DNA and RNA in the Supernatant

According to the literature [27], *C. acnes* in the logarithmic phase was treated with OCM at 1*MIC and 2*MIC for 6 h. The supernatant was filtered through a 0.22-μm microporous filter and analyzed with a Nanodrop 2000 (Thermo Fisher Scientific, Waltham, MA, USA) to determine the release of DNA and RNA (measured at 260 nm and 230 nm).

### 3.7. Morphological Analysis

The effect of OCM on the morphology of *C. acnes* can be observed using SEM and TEM according to the described method [28]. Briefly, the logarithmic phase of *C. acnes* was resuspended in the fresh medium of ~10^8^ CFU/mL and incubated with OCM of 1*MIC, 2*MIC, and 4*MIC at 37 °C for 6 h. After incubation, the bacteria were collected by centrifugation, washed three times, and fixed overnight. Subsequently, the samples were dehydrated with ethanol. The dehydrated samples were dried for 2 h using a critical point dryer. Finally, the *C. acnes* were thinly sputtered with a thin layer of gold, and observations were conducted using SEM (SU8020, Hitachi, Tokyo, Japan). The samples for TEM were prepared following the same procedure as for SEM. Bacterial cells were fixed overnight, washed three times, dehydrated with ethanol, embedded overnight, cut into ultrathin sections, stained, and finally observed by TEM (Hitachi H-7650, Tokyo, Japan).

### 3.8. Metabonomics Studies

#### 3.8.1. Preparation and GC–MS Analysis of Metabolites

The GC–MS was utilized to analyze the metabolites of *C. acnes*. In short, logarithmic bacteria (~10^8^ CFU/mL) were treated with OCM (1*MIC). The bacteria were collected by centrifugation, washed in prechilled PBS (4 °C), and then subjected to ultrasonication. The conditions included sonication for 2 s, pausing for 3 s, continuously for 20 min, and ultrasound power set at 320 W. Following centrifugation, 8 µL of ribitol was added to the supernatant (approximately 0.8 mL) as the internal standard. Subsequently, samples were derivatized using MSTFA and analyzed by GC–MS (Agilent 7890A GC, Palo Alto, CA, USA) as previously described [29,30,31]. In the splitless injection mode, the injection volume was 1 µL. The heating steps were as follows: initial temperature of 70 °C, maintained for 3 min, then heated at a rate of 5 °C/min to 285 °C, followed by heating at a rate of 20 °C/min to 310 °C, and maintained at 310 °C for 7 min. The temperature of the ion source was 230 °C, and the temperature was set at 150 °C. Helium was used as the carrier gas with a flow rate of 1.0 mL/min.

#### 3.8.2. Analysis of Metabolomics Data

After peak recognition and deconvolution, metabolites were identified using NIST and Fiehn databases. Mass spectrometry data were normalized for subsequent analysis using internal standards. Based on the multiple changes compared to the control group, a combination of *p*-values from the standardized peak area two-tailed Student *t*-test and adjusted *p*-values after correction were used to select differential metabolites. PCA and OPLS-DA were performed to visualize metabolic changes. Finally, the enrichment analysis was conducted using MBROLE 2.0 software (https://csbg.cnb.csic.es/mbrole2/, accessed on 7 June 2023) with the identified differential metabolites, and the top 20 pathways were extracted for visualization.

### 3.9. Modified Proteomics

#### 3.9.1. Proteins Extraction and Digestion

The bacteria were treated with OCM (0.5*MIC), and the culture and treatment process of the bacteria were the same as described in Section 3.7 “Morphological Analysis”. The total protein was quantified using the BCA protein assay kit. The supernatant was precipitated with ice-cold acetone (1:4, *v*/*v*) overnight at 4 °C. It was then centrifuged at 8000× *g* for 30 min at 4 °C to collect the precipitate, which was washed successively with 500 μL of pre-cooled acetone, pre-cooled 70% ethanol, and pre-cooled acetone. The pellet was dried and resuspended in 50 μL of UA buffer (8 M Urea, 0.1 M Tris/HCl, pH 8.5) supplemented with 2 μL of 1,4-dithiothreitol (final concentration of 2 mM) and then incubated at 30 °C for 1.5 h. After adding 13 μL of 50mM iodoacetamide, the samples were kept in the dark for 40 min and then diluted to 600 μL with 50 mM NH_4_HCO_3_. Subsequently, trypsin solution (0.25 μg/μL) was added to digest samples at a mass ratio of 1:60 (trypsin/protein) for 12 h at 37 °C. Desalting was then carried out using a C18 stage-tip column, and all labeled peptide products were pooled and vacuum-dried.

#### 3.9.2. Protein Identification and Data Analysis

The hydrolyzed peptide was separated and analyzed using nano LC–MS/MS analysis. The chromatographic conditions involved reversed-phase columns (Reprosil-Pur C18-AQ, 75 μm × 30 cm, 2 μm) with a flow rate of 200 nL/min. The mobile phase consisted of 0.1% formic acid and 80% acetonitrile with a linear gradient elution of 0.1% formic acid. MS analysis spectra were obtained using a Q-Exactive Plus mass spectrometer (Thermo Scientific, USA). Full scans were conducted in the mass range of 355–1700, and MS/MS scans were performed at a resolution of 35,000. Proteome Discoverer was utilized for protein searching. A total of 34,899 proteins of *C. acnes* were downloaded from the UniProt database on 18 May 2023. According to the literature, the parameters were set as follows: trypsin digestion with up to two missed cleavages and urea methylation of cysteines as a fixed modification. Additionally, cinnamaldehyde and dehydrated cinnamaldehyde modifications were considered variable modifications. The modified molecular compositions for cysteine, aspartate, histidine, and lysine were Cin1: +132.16 Da and Cin2: +114.16 Da. Peptide spectral matches were validated based on *p*-values at a 1% false discovery rate.

### 3.10. The Assay of NADH, NAD^+^, ATP Content, NADH Dehydrogenase and Malate Dehydrogenase Activity

We inoculated *C. acnes* cultured to the logarithmic phase (1 × 10^9^ CFU/mL) onto OCM containing different mass concentrations (200, 400, and 800 μg/mL) of BHI medium. The bacterial solution was cultured under anaerobic conditions at 37 °C and 180 rpm for 8 h before centrifugation to obtain the supernatant. The NAD, NADH, ATP content, NADH dehydrogenase, and malate dehydrogenase activity were measured using a reagent kit.

### 3.11. Changes in Amino Acid and Its Analog Content over Time

The metabolite changes over time were studied through GC–MS analysis. The bacteria were treated with OCM (1*MIC), and the treatment process of the bacteria was the same as described in Section 3.7 “Morphological Analysis”. The content of tromethamine, glutamic acid, *N*,*N*-dimethylglycine, glycine, lysine, ornithine, proline, threonine, and serine were detected from 0 to 8 h.

### 3.12. Statistical Analysis

Unless otherwise specified, all statistical analyses were conducted in GraphPad Prism (version 8.0.2.263). All the experiments were in triplicate, with at least three biological replicates. The data were analyzed by one-way analysis of variance (ANOVA) and expressed as the mean ± standard error of the mean (mean ± SEM). *p* ≤ 0.05 were regarded to be significant (* *p* ≤ 0.05; ** *p* ≤ 0.01; *** *p* ≤ 0.001; **** *p* ≤ 0.0001 vs. control group).

## 4. Conclusions and Prospect

OCM is a volatile component extracted from the branches and leaves of *Cinnamomum*, a plant in the *Lauraceae* family. It is a natural and efficient fungicide with significant potential in food, medicine, and cosmetics.

However, the target of OCM antibacterial activity needs to be clearly explained. This article utilizes advanced chromatography and high-resolution mass spectrometry technology to investigate the target of OCM on *C. acnes*.

The previously reported antibacterial mechanisms of cinnamaldehyde include aldehyde poisoning and acid stress, interference with carbohydrate biosynthesis, disruption of energy metabolism, increased reactive oxygen species (ROS) levels, and disturbance in the energy metabolism and TCA cycle. In this study, cinnamaldehyde was successfully detected in OCM covalently modified NADH quinone oxidoreductase, ABC transporter, and ATP binding protein of *C. acnes*. It has been confirmed that OCM affects the glucose and proline metabolism of *C. acnes*, disrupts the balance between NADH and NAD^+^, and hinders ABC transport. This research offers potential targets for the development of antibacterial drugs and therapies.

NADH is an essential coenzyme that is vital in biological redox reactions. Elevated concentrations of NADH can stimulate the generation of ROS. Cinnamaldehyde covalently modifies NADH-quinone oxidoreductase, disrupting the NADH–NAD^+^ cycle, inevitably affecting critical physiological functions such as cell phosphoglycolysis, energy metabolism, carbohydrate biosynthesis, and disrupting cell signal transduction and gene expression. Cinnamaldehyde covalently modifies the ABC transporter: ATP binding protein, which can limit the energy supply in the ABC transporter system and affects cell transport of ions, amino acids, nucleotides, polysaccharides, peptides, and other substances. Cinnamaldehyde covalent modification of enoyl CoA hydroxylase/isomerase family protein affects cellular fatty acids β-oxidation in the oxidative metabolic pathway. Covalently modified enolase affects the formation of high-energy compound phosphoenolpyruvate from 2-phosphoglycerate and thus limits cellular glycolysis.

In summary, we found that inhibiting *C. acnes* of cinnamaldehyde in the OCM is consistent with the previously reported mechanism of OCM inhibiting *Escherichia coli*, *Staphylococcus aureus*, and methicillin-resistant *Staphylococcus epidermidis*. The results suggest that cinnamaldehyde in OCM covalently modifies NADH quinone oxidoreductase, ABC transporter, and ATP-binding protein, leading to direct cell death.

The target of OCM inhibiting *C. acnes* is clear. There is sufficient evidence for covalent binding of α,β-unsaturated aldehydes with the target proteins. Additionally, our research team is investigating the dehydration covalent modification of proteins containing adjacent cysteine histidine, as well as adjacent cysteine and aspartic acid with α,β-unsaturated aldehydes. This research lays the groundwork for the application of α,β-unsaturated aldehydes as covalent inhibitors in the pharmaceutical field.

## Figures and Tables

**Table 1:** The main components and their relative contents (%) of the OCM by GC–MS analysis.

Peak	RT (min)	Structural Formula	Name	Percentage (%)	Classification
1	10.414	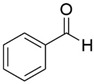	Benzaldehyde	1.276	Aromatic aldehyde
2	14.143	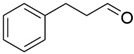	Benzenepropanal	2.122	Phenylpropanoids
3	14.397	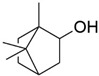	Bornanol	0.312	Monoterpenoids
4	14.789	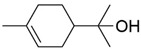	α-Terpineol	0.112	Monoterpenoids
5	15.259	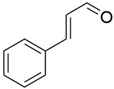	(Z)-Cinnamaldehyde	0.871	Phenylpropanoids
6	15.456	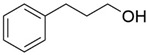	3-Phenylpropanol	0.318	Phenylpropanoids
7	16.64	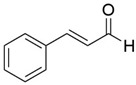	Trans-Cinnamaldehyde	85.308	Phenylpropanoids
8	17.039	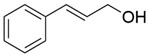	Cinnamyl alcohol	0.795	Phenylpropanoids
9	18.317	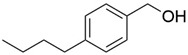	4-Butylbenzyl alcohol	0.213	Aromatic alcohol
10	18.628	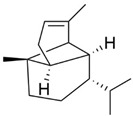	α-Copaene	0.250	Monoterpenoids
11	19.633	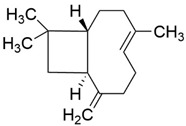	β-Caryophyllene	0.200	Bicyclic sesquiterpanes
12	19.97	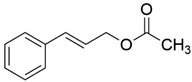	Cinnamyl acetate	0.571	Phenylpropanoids
13	21.424	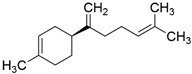	β-Bisabolene	0.267	Sesquiterpenoids
14	21.702	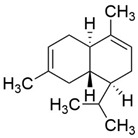	β-Cadinene	0.217	Sesquiterpenoids
15	21.858	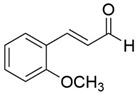	2-Methoxycinnamaldehyde	2.145	Phenylpropanoids
16	22.121	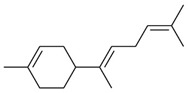	α-Bisabolene	0.191	Sesquiterpenoids
17	23.062	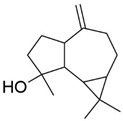	Spathulenol	0.347	Sesquiterpenoids
18	23.66	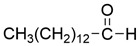	Tetradecanal	0.302	Aliphatic compound
19	25.352	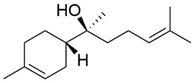	a-Bisabolol	0.396	Sesquiterpenoids
Total			identified	96.213	

## Data Availability

All data are included in the manuscript.

## Abbreviations

OCM—*Oleum cinnamomi*; *C. acnes*—*Cutibacterium acnes*; GC–MS—gas chromatography–mass spectrometry; LC–MS—liquid chromatography–mass spectrometry; ATP—adenosine triphosphate; CFU—colony forming units; SEM—scanning electron microscopy; TEM—transmission electron microscope; MIC—minimum inhibitory concentration; MBC—minimal bactericidal concentration; NIST—National Institute of Standards and Technology; TCA—tricarboxylic acid; MDH—malate dehydrogenase; NADH—nicotinamide adenine dinucleotide; NAD^+^—nicotinamide adenine dinucleoside phosphorus; ROS—reactive oxygen species; KEGG—Kyoto Encyclopedia of Genes and Genomes; BHI—brain heart infusion broth; RCA—reinforced Clostridium agar; OD—optical density; DNA—deoxyribonucleic acid; RNA—ribonucleic acid; MSTFA—N-methyl-N-trimethylsilyltriflfluoroacetanamide; PCA—principal component analysis; OPLS-DA—orthogonal partial least squares discriminant analysis; FC—fold change; p.adj—*p*-value adjusted; EMP—glycolysis; HMP—hexose monophosphate pathway.
